# Field study to investigate the effectiveness and safety of a novel orally administered combination drug product containing milbemycin oxime and lotilaner (Credelio^®^ Plus) against natural flea and tick infestations on dogs presented as veterinary patients in Europe

**DOI:** 10.1186/s13071-021-04808-0

**Published:** 2021-06-04

**Authors:** Sophie Forster, Scott Wiseman, Daniel E. Snyder

**Affiliations:** 1 Elanco Animal Health, Bartley Way, Bartley Wood Business Park, Hook, Hants RG27 9XA UK; 2 Daniel E. Snyder, DVM PhD. Consulting, LLC, Indianapolis, IN 46229 USA

**Keywords:** Credelio Plus^®^, Dog, Effectiveness, Fleas, Lotilaner, Milbemycin oxime, Oral, Ticks, Veterinary patients

## Abstract

**Background:**

A pivotal randomised, blinded, positive-controlled, multicentre, European field study was conducted to evaluate the effectiveness and safety of a novel combination tablet of lotilaner and milbemycin oxime (Credelio^®^ Plus) administered orally to client-owned dogs naturally infested with fleas and/or ticks.

**Methods:**

In this field study, households with flea- or tick-infested dog(s) were enrolled on Day 0 into the study to provide data for either the tick or flea infestation cohorts. Households were randomised in a 2:1 ratio to receive either the combination investigational product (IP, Credelio Plus^®^ tablets) or the control product (CP: Nexgard Spectra^®^ tablets). Dogs were administered IP (flea cohort *n* = 135; tick cohort: *n* = 147) or CP (flea cohort: *n* = 67; tick cohort: *n* = 74) once every 4 weeks for a total of three times at a dose rate of 20.0–41.5 mg/kg bodyweight lotilaner and 0.75–1.53 mg/kg bodyweight milbemycin oxime (IP) or as recommended (CP). Percentage reduction was calculated by comparing individual dog flea and tick counts at each assessed post-treatment time point to their respective baseline (pre-treatment) infestation. Resolution of the clinical signs of flea allergy dermatitis (FAD) was assessed in flea-allergic dogs on the days that flea counts were performed.

**Results:**

Flea effectiveness of Credelio Plus^®^ after 3 consecutive monthly treatments was 100% against *Ctenocephalides felis*, *C. canis* and *Pulex irritans*. Tick effectiveness of Credelio Plus^®^ over the same time frame was 99.3% for *Ixodes ricinus* and 100% against *Rhipicephalus sanguineus* (*s.l.*). Flea effectiveness of the CP after three consecutive monthly treatments was 100% against *C. felis*, *C. canis* and *P. irritans*. Tick effectiveness of the CP over the same time frame was 99.8% for *I. ricinus* and 100% against *R. sanguineus*. Credelio Plus^®^ was well tolerated based on the safety assessments in all treated dogs in this field study. Within both treatment groups there was a reduction in total FAD scores from baseline.

**Conclusions:**

This pivotal European field study demonstrated the excellent effectiveness and safety of a combination of lotilaner and milbemycin oxime (Credelio Plus^®^) administered orally to dogs naturally infested with fleas and/or ticks.

**Graphic Abstract:**

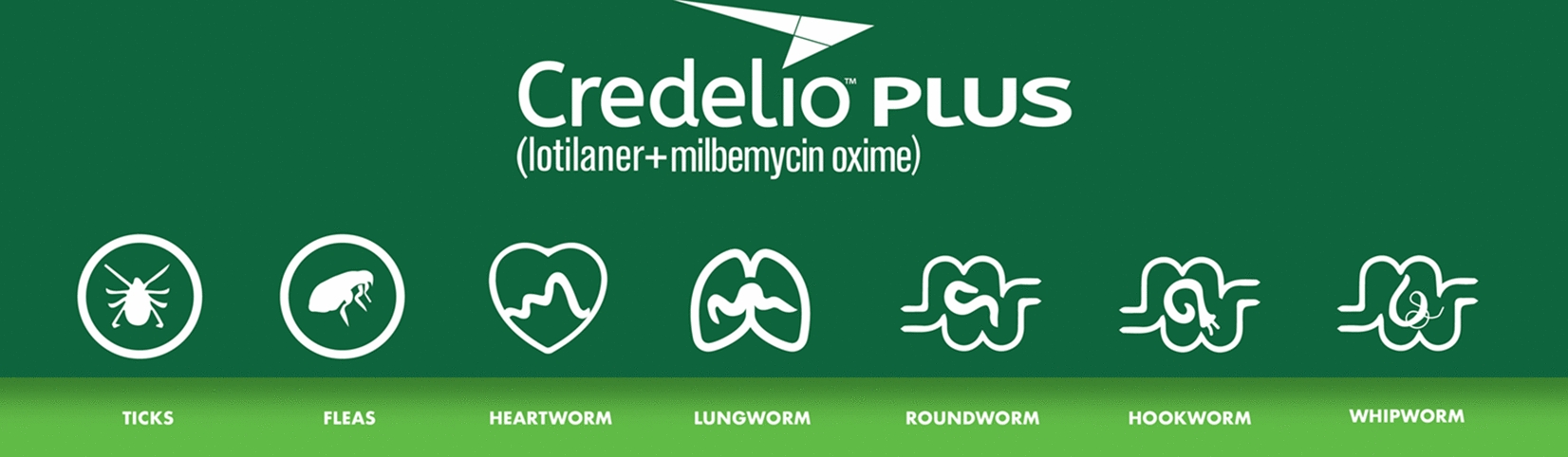

## Background

In Europe, the cat flea (*Ctenocephalides felis*) is the species most commonly found on dogs; however, in certain regions the dog flea (*Ctenocephalides canis*) and the hedgehog flea (*Archaeopsylla erinacei*) are also routinely seen [1]. Blood-feeding by *C. felis* and *C. canis* can cause local irritation known as flea bite dermatitis and with repeated exposure dogs may develop flea allergy dermatitis (FAD) [2, 3]. Infestations with large numbers of cat or dog flea species can lead to anemia, which can be more pronounced in young or debilitated animals [2]. Fleas are known to transmit several zoonotic disease agents such as flea-borne spotted fever (*Rickettsia felis*), murine typhus (*Rickettsia typhi*) and cat scratch disease (*Bartonella henselae*) [4, 5]. Fleas additionally serve as intermediate hosts for helminths including the dog tapeworm *Dipylidium caninum* [6].

Ticks are found throughout most of Europe and on dogs predominantly include species from the genera *Dermacentor*, *Ixodes* and *Rhipicephalus* [1]. Tick blood-feeding causes nuisance to the dog and their owner and skin irritation at the site of attachment, and heavy infestations can result in anaemia [1, 7]. In Europe tick-transmitted disease agents, some of which are zoonotic, include *Babesia canis* vectored by *Dermacentor reticulatus,* canine and human anaplasmosis (*Anaplasma phagocytophilum*) and Lyme borreliosis (*Borrelia burgdorferi* (*s.l.*)), both of which are transmitted by *Ixodes ricinus* [5]. Ticks in the genus *Rhipicephalus* are known to transmit canine ehrlichiosis (*Ehrlichia canis*), babesiosis (*Babesia vogeli*, *Babesia gibsoni*), hepatozoonosis (*Hepatozoon canis*) and Mediterranean spotted fever (*Rickettsia conorii*) [8].

It is recommended by global veterinary practice guidelines (e.g. ESCCAP) that dogs exposed to flea and tick infestations in endemic regions of Europe and their potential for disease transmission should be prescribed approved pulicidal and acaricidal products to provide consistent and ongoing efficacious flea and tick treatments [1, 9]. Lotilaner is an ectoparasiticide and is one of several similar active ingredients that contain this newer class of chemistry called the isoxazolines [10]. Lotilaner was previously developed as an oral monthly administered chewable tablet for use in dogs and cats as a mono-use drug product (Credelio™, Elanco Animal Health). Credelio™ has been shown to provide fast and consistent month-long effectiveness against fleas and ticks in dogs and cats [11–23]. It was also shown to have effectiveness against *Demodex* spp. mites in dogs [24]. To provide broad-spectrum adult and larval endoparasite effectiveness, in addition to flea, mite and tick prevention and treatment in dogs, an oral chewable tablet formulation combination drug product (Credelio Plus^®^) was developed that contains a minimum dose of 0.75 mg/kg (range 0.75–1.53 mg/kg) of milbemycin oxime (MO) and a minimum dose of 20 mg/kg (range 20–41.5 mg/kg) of lotilaner.

In this publication we report the effectiveness and safety of the novel lotilaner and MO combination investigational product (IP), Credelio Plus^®^, compared to an authorised control product (CP), NexGard Spectra^®^, for the prevention, treatment and control of naturally occurring flea and tick infestations in client-owned dogs in Europe under field conditions.

## Methods

The study was a pivotal randomised, blinded, positive-controlled, multicentre, GCP-compliant, European field study to evaluate the effectiveness and safety of a combination of lotilaner and MO chewable tablets (IP) administered orally to dogs naturally infested with fleas or ticks. Dogs were enrolled from 25 veterinary clinical sites located in Germany (*n* = 12), Hungary (*n* = 6) and Spain (*n* = 7), covering different climate zones across Europe. The veterinary practices were selected in geographic areas where flea and tick prevalence was known to be high. This field study was conducted in accordance with the World Association for the Advancement of Veterinary Parasitology (WAAVP) guidelines for evaluating the effectiveness of parasiticides for the treatment, prevention and control of flea and tick infestation on dogs and cats, VICH Guideline 9, Guideline on Statistical Principles for Veterinary Clinical Trials and Guideline for the Demonstration of Effectiveness of Ectoparasiticides [25–28]. Study site personnel involved in making assessments of flea or tick effectiveness and safety were masked to treatment assignments. Day 0 was defined as the day the first treatment was administered to each enrolled dog.

### Animals

Dogs considered for enrolment had to be at least 8 weeks of age and weigh ≥ 2.0 kg on Day 0. To be enrolled in the study, on Day 0 the owner was required to complete and sign an Owner Consent Form and to provide the dog’s prior and current medical history. Dogs also were required to be negative on Day 0 to a HW antigen test (using a commercial SNAP antigen assay) that was performed by the investigator. Blood samples collected on Day 0 that were negative on the HW antigen test were further examined for HW microfilaria (Modified Knott’s test) as well as real-time PCR (speciation of microfilaria if found) at the designated clinical laboratory. Dogs that were positive for HW microfilaria were removed from the study once these results were submitted to the investigator.

Single- and multi-dog (maximum of 3 dogs per household) households were eligible for participation. The first dog of a household with three or more live, attached ticks or five or more fleas was enrolled as the primary dog, with up to two additional dogs in the household enrolled as supplementary dogs. If a primary dog fulfilled both the inclusion criteria of a flea-infested patient and a tick-infested patient, that primary dog was enrolled in the tick population. All supplementary dogs in a household had only physical examinations and body weights that were collected during the study. The product effectiveness of the IP or CP was evaluated only in each enrolled primary dog, and the individual primary dog was considered the experimental unit assessed for product effectiveness. Households were enrolled into the study to provide data for either the tick infestation or flea infestation cohort of the study; no household was enrolled into both cohorts. All households were randomised to one of the two treatment groups, IP or CP. The IP and CP products, route, dosage and frequency of treatment were the same in both cohorts of the study.

Dogs of any breed and sex and reproductively neutered or intact (non-pregnant and non-lactating if female) and not intended for breeding during the study were eligible for enrolment. Other animal eligibility criteria included: the dog was of a suitable temperament (not fractious); the dog was owned by a client, *not* an investigator or the clinic’s veterinarian(s), staff or relatives thereof; no other dogs in the household were participating in this study; if housed primarily outdoors, the dog was maintained in a controlled home/yard environment that allowed for observations required by the study; the dog was generally healthy, i.e. expected to survive the study duration based on history and physical examination; the dog was free of a serious disease that would interfere with the objectives of the study. If serious or systemic disease was present (e.g. epilepsy, diabetes mellitus, hypothyroidism, etc.), it was to be diagnosed accordingly and well controlled with the appropriate ongoing treatments that were anticipated to continue throughout the entire study period for the specific disease state. Dogs in each enrolled household were prohibited to use any commercial product with effectiveness against ticks and/or fleas on the animal or its home environment during the course of this field study.

The owner returned the primary dog and supplementary dogs to the clinic for various procedures and assessments including weekly for flea or tick counts, biweekly for FAD assessments or monthly for treatment until the end of the live phase on Study Day 84 ± 2.

### Randomisation

A randomisation algorithm within the electronic data capture system employed in this study was used to assign each household with an enrolled primary dog to Group 1 (IP; Credelio Plus^®^ tablets; MO + Lotilaner) or Group 2 (CP; Nexgard Spectra^®^ tablets; afoxolaner + milbemycin oxime). The randomisation was stratified by site to ensure each site had households allocated to both IP and CP treatment groups. Households were blocked in a 2:1 ratio (IP:CP; block size of 3). Households were randomised in the sequence of enrolment. All dogs from the same household were allocated to the same treatment. The examining veterinarian was not aware of the allocation of the treatment to each enrolled dog.

### Treatment

Dogs were administered IP or CP once every 4 weeks for a total of three times; on Study Day 0 and subsequently on Study Days 28 ± 2 and 56 ± 2. A designated unmasked dispenser at each veterinary clinic was solely responsible for dispensing of the experimental IP and CP products and giving and reviewing product administration instructions to the owners. The IP, Credelio Plus^®^ combination tablets were supplied in five different strengths. This allowed for the targeted dose range of the flavored tablet dosage form for the IP and a unit dose reflecting the intended oral treatment at approximately 20–40 mg lotilaner per kg body weight and approximately 0.75–1.5 mg milbemycin oxime per kg body weight. The CP, Nexgard Spectra^®^ tablets (afoxolaner and milbemycin oxime; Boehringer Ingelheim) were dispensed per label directions.

The owner was instructed on storage and administration of the assigned product while at the veterinary clinic and then administered the tablets at home and confirmed product consumption. Each product was given under the following conditions to ensure maximum product effectiveness: the IP was to be administered to the dog under fed condition (last meal taken approximately 30 min prior to treatment). If the dog had not been fed approximately 30 min prior to treatment, or in the case of doubt, food was offered to the dog (the targeted amount was one third of the daily ration) prior to treatment; the CP was given as per label instructions.

### Flea and tick counts

Flea counts on primary dogs were conducted pre-treatment on Day 0 and post-treatment on Days 14 ± 2, 28 ± 2, 56 ± 2 and 84 ± 2. The entire hair coat on each dog was systematically combed using an extra-fine-tooth flea comb until all fleas were removed. The veterinary clinic staff conducting the flea counts had been trained on this procedure. Each dog was combed for a minimum of 10 min. If fleas were found in the last minute, then combing was continued for an additional 5 min. Fleas capable of maintaining upright orientation and/or coordinated movement were considered alive. All live fleas were counted and recorded as they were removed. On each day fleas were collected from a primary dog, they were stored for subsequent speciation.

Tick counts on primary dogs were conducted pre-treatment on Day 0 and post-treatment on Days 7 ± 1, 14 ± 2, 21 ± 2, 28 ± 2, 42 ± 2, 56 ± 2, 70 ± 2 and 84 ± 2 and recorded. For each dog, the hair coat of the entire body was manually examined so that the skin and any attached ticks were exposed. As the integrity of the ticks (head and mouthparts of the tick were intact and attached to the body) needed to be maintained to allow for tick identification, the use of a commercial tick removal hook was suggested; however, other standard veterinary methods that allowed for intact tick removal could also be used. Prior to tick removal, the viability was assessed by gently probing the tick (e.g. touching with a pen or blowing on tick) to assess if the legs moved, in which case the tick was considered viable. On each day attached ticks were collected and removed from a primary dog, they were stored (separately for live, attached and dead, attached ticks) for subsequent speciation.

Fleas and ticks stored from each primary dog were sent on the day of collection to Vet Med Labor GmbH, IDEXX Bioresearch, Ludwigsburg, Germany. The determination of species, stage (larva, nymph, adult) and sex (for adult ticks only) was performed in accordance with the laboratory’s standard procedures and recorded based on morphological characteristics and keys for each tick species that was identified.

### Assessment of flea allergy dermatitis (FAD)

Clinical signs associated with flea allergy dermatitis (FAD) were evaluated pre-treatment on Day 0 and post-treatment on Days 14 ± 2, 28 ± 2, 56 ± 2 and 84 ± 2 for both the IP and CP flea cohorts. Post-treatment FAD assessments were performed on primary flea cohort dogs that presented with a total FAD score ≥ 1 on Day 0. Clinical signs of FAD assessed were pruritus, erythema, scaling, papules, alopecia and dermatitis/pyodermatitis. The examining veterinarian assigned and recorded a score on the Flea Allergy Dermatitis Assessment Form. The scoring system for FAD signs were: absent, mild, moderate and severe with numerical values 0, 1, 2 and 3 assigned, respectively. For the sign “pruritus” the scoring was done as follows: 0 = none/no scratching; 1 = mild/occasionally scratching; 2 = moderate (frequently scratching and/or biting itself); 3 = severe (intense scratching/biting itself). The numerical values were used in the statistical analysis and a total FAD score was calculated for each dog at each time point as the sum of the clinical sign scores.

### Safety assessments

The safety assessment was performed for all dogs that had received at least one treatment (primary and supplementary dogs) with either IP or CP. During the study period from the day of first treatment administration (Day 0) to study completion (Day 84 ± 2), study dogs treated with the IP or CP were observed regularly. Physical examinations and body weights were performed on each primary dog at the time of each of the flea or tick assessment time points. Blood samples were obtained for haematology and serum chemistry assessments on Day 0 (pre-treatment) and on Day 84 ± 2 for both the primary and supplementary dogs. AEs and concomitant treatment(s) for the primary and supplementary dogs were also recorded, as necessary. Supplementary dogs did not undergo flea or tick counts or FAD assessment.

### Data analysis

The enrolled primary dog in each household was considered the experimental unit for flea or tick counts and FAD assessments for the IP and CP. Analysis of safety data included all dogs receiving either IP or CP, irrespective of whether the household was enrolled for the flea or tick infestation cohort of the study. Separate analysis populations were defined for the assessment of safety, flea, tick and FAD effectiveness data. The 5% level of significance (*p* < 0.05 for two-sided tests) was used in all statistical testing.

The total live flea count (i.e. all flea species combined), as recorded by the Investigator, was the primary variable for assessment of effectiveness against flea infestations. The total live tick count (i.e. all tick species combined), as recorded by the investigator, was the primary variable for assessment of effectiveness against tick infestations. Count data were transformed using ln(count + 1) to account for the asymmetric, skewed nature of these data and to allow for zero counts. The transformed data were analysed using repeated measures mixed model methodology. Treatment group, time (Study Day) and time-by-treatment group were fixed effects in the model. Site and site-by-treatment interaction were fitted as random effects. Dog was also fitted as a random (repeated measures) effect to account for the correlation between observations on the same dog. Different covariance structures were investigated to model the correlation between observations on the same dog, with the structure in the final model being selected based on the lowest Akaike information criterion (AIC) value. All statistical analyses used the statistical package SAS 9.4. The percent (%) reduction in flea or tick counts was calculated using back-transformed model LS means with the following equation: [(C − T)/C] × 100, where C is the mean pre-treatment live parasite count and T is the mean post-treatment live parasite count. The calculation was repeated using arithmetic means (AM). In addition, the 95% confidence interval for the count ratio (IP:CP) post-treatment was formed. In case the percent reduction in either group fell below the 95% (fleas) or 90% (ticks) thresholds, the difference in success rates was also evaluated. For each dog, success was defined as a percentage reduction from baseline of at least 90% (ticks) or 95% (fleas). A 95% confidence interval was then calculated on the difference of the success rate between IP and CP. If the lower bound of the interval was > –15%, then non-inferiority was accepted. Non-inferiority of the IP in relation to the CP was assessed. Non-inferiority was declared if the following conditions were met: (1) the decrease in least squares (LS) mean count post-treatment was statistically significant (*p* < 0.05) compared to pre-treatment for each of the IP and CP groups and (2) the reduction in count post-treatment, based on LS (geometric means), was ≥ 95% (fleas) or ≥ 90% (ticks) for both the IP and CP. Summary statistics for the live flea, live tick and dead tick counts were also provided to support the statistical analysis. The summary statistics included arithmetic mean, geometric mean, median, standard deviation, minimum and maximum.

The total FAD score (defined as the sum of the component scores) was analysed using analysis of covariance (ANCOVA) with treatment group, time point and treatment-by-time interaction as fixed effects. Baseline FAD and baseline FAD-by-time were fitted as covariates. Site and site by treatment interaction were fitted as random effects. The 95% confidence intervals for the difference between treatment groups were formed and non-inferiority assessed with a 15% limit. Summary statistics for the total score and for each individual component were also presented.

## Results

### Primary dog demographics

A total of 429 dogs were screened and recruited from 25 veterinary clinical sites located in Germany (*n* = 12), Hungary (*n* = 6) and Spain (*n* = 7). These 429 primary dogs were randomised to IP or CP groups, with 423 dogs (Table 1) included in this population for analysis since 6 enrolled dogs were excluded from the flea and tick effectiveness populations. Descriptive statistics on dog age, sex, body weight on Day 0, breed and coat length are summarised in Table 1.

**Table 1:** Demographics of primary dogs enrolled and included in flea and tick effectiveness populations in European field study to evaluate the effectiveness of Credelio Plus® against natural flea and tick infestations

Demographic	Flea cohort		Tick cohort	
	Credelio Plus^®^ (*n* = *135*)	Afoxolaner + MO (*n* = *67*)	Credelio Plus^®^ (*n* = *147*)	Afoxolaner + MO (*n* = *74*)
Purebred (*n*) (%)	73 (54.1)	40 (59.7)	88 (59.9)	46 (62.2)
Mixed breed (*n*) (%)	62 (45.9)	27 (40.3)	59 (40.1)	28 (37.8)
Age, mean (months)	65.7	68.8	68.2	73.1
Age, range (months)	2–174	2–162	2–210	4–198
Age group < 12 months (*n*) (%)	22 (16.3)	11 (16.4)	14 (9.5)	5 (6.8)
Age group ≥ 12 months (*n*) (%)	113 (83.7)	56 (83.6)	133 (90.5)	69 (93.2)
Body weight, mean (kg)	16.4	17.2	20.0	19.3
Body weight, range (kg)	2.3–63.3	3–45.3	2.9–58.3	2.8–53.3
Male (*n*) (%)	66 (48.9)	41(61.2)	75 (51)	34 (46)
Female (*n*) (%)	69 (51.1)	26 (38.8)	72 (49)	40 (54)
Coat length				
Short	59	34	69	41
Medium	53	20	60	23
Long	23	13	18	10

#### Demographics and evaluable flea population

A total of 138 primary dogs treated with IP and 67 primary dogs treated with CP were enrolled into the flea cohort for the study. Most of these dogs (IP: 135, 97.8%; CP: 67, 100%) had no major protocol deviations and were therefore included in the Flea Effectiveness population for analysis.

#### Demographics and evaluable FAD population

From the animals enrolled into the flea cohort a total of 111 primary dogs treated with IP and 55 primary dogs treated with CP had an FAD score ≥ 1 on Day 0. Most of these dogs (IP: 110, 99.1%; CP: 55, 100%) had no major protocol deviations and were therefore included in the FAD analysis.

#### Demographics and evaluable tick population

A total of 149 primary dogs treated with IP and 75 primary dogs treated with CP were enrolled into the tick cohort of the study. Most of these dogs (IP: 147, 98.7%; CP: 74, 98.7%) had no major protocol deviations and were therefore included in the Tick Effectiveness population for analysis.

#### Demographics and evaluable safety population

The safety population consisted of all dogs that received at least one dose of either IP (*n* = 427, 99.3%) or CP (*n* = 212, 99.5%). Both primary and supplementary dogs from the flea and tick portions of the study were included in this population.

The primary dog descriptive statistics summarised in Table 1 on dog age, sex and body weight on Day 0 illustrate that treatment groups had comparable baseline characteristics regarding age, body weight on Day 0, purebred/mixed bred distribution and percentages of dogs with short, medium or long coat length. The female/male distribution was less similar but it was anticipated that this had minimal to no impact on response as no sex effect has been observed in previous effectiveness studies using Credelio Plus^®^.

### Safety

During the course of the Day 84 ± 2 study period, all abnormal events, regardless of their causality, duration,or severity, were recorded for each enrolled dog. Overall, the % animal rate was slightly higher in the CP group. The animal rate was 6.6% for dogs in the CP group and 4.9% for dogs in the IP group that had adverse events. The majority of these adverse events (AEs) were categorised as non-serious and were not related to treatment with either IP or CP. Most non-serious AEs in the IP group affected the digestive tract (diarrhoea, emesis and nausea) followed by skin disorders (bacterial infections, dermatitis and eczema, pruritus and skin lesions) and systemic disorders (discomfort, lethargy and trauma). In the CP group, AEs most commonly affected the digestive tract (diarrhoea and emesis) and the skin (bacterial infections, dermatitis and eczema and pruritus) followed by the eyes (protruding third eye lid), systemic health (lethargy) and others (uncoded sign: tapeworm infection). Serious adverse events were documented for ten dogs (6 IP dogs, 4 CP dogs). Each study site investigator assessed most of these observed serious adverse events as unrelated to the IP or CP treatments based on clinical examinations, history and timing of each event.

Post-treatment haematology and serum chemistry were generally unremarkable and similar between treatment groups. Although individual animals had individual out of range parameters or changes considered clinically significant by the investigator in haematology or clinical chemistry parameters at Day 84 in both groups, none showed a clinically relevant difference at the population level. All mean values remained within normal reference ranges.

Summary statistics for body weight, change from baseline body weight (first visit on Day 0 to final visit on Day 84) and percentage change from baseline body weight at each visit were assessed by treatment group for dogs < 12 months and dogs ≥ 12 months. There were no significant differences in body weights between treatment groups in the safety population at any visit or over the entire study period.

A number of concomitant medications or vaccinations were given to dogs in both the IP and CP treatment groups. Vaccines were most frequently and concurrently administered in both treatment groups. Concurrent treatments used during the study included licensed animal drugs, human drugs used off-label, alternative/herbal remedies, medicated shampoos or other topical treatments and prescription diets. There were no adverse events associated with the concomitant use of these treatments and thus both the IP and the CP were considered to be well tolerated and used safely with numerous concomitant treatments and vaccines that are routinely administered to dogs in veterinary medicine.

### Effectiveness

#### Flea cohort

The results for all identified flea species combined are summarised in Table 2. Pre-treatment arithmetic mean live flea counts were 11.8 (range 5–275) in the Credelio Plus^®^ group and 11.4 (range 5–139) in the afoxolaner + milbemycin oxime group. Compared to pre-treatment, on Days 14, 28, 56 and 84 mean live flea counts were reduced by 98.7%, 99.6%, 100% and 100%, respectively, in the Credelio Plus^®^ group. In the afoxolaner + milbemycin oxime group, mean live flea counts were reduced by 98.5%, 97.2%, 99.6% and 100%, respectively. Post-treatment flea counts on Days 14, 28, 56 and 84 were significantly lower than baseline counts in both treatment groups (*p* < 0.001). The effectiveness of Credelio Plus^®^ was non-inferior to that of NexGard Spectra^®^ at all post-treatment time points.

**Table 2:** Effectiveness of three consecutive monthly doses of Credelio Plus^®^ and afoxolaner + milbemycin oxime based on investigator counts of natural flea infestations for all flea species on client owned dogs in European field study

Study day	Treatment group	*n*	Live flea counts		% Effectiveness
			Range	Arithmetic mean	
0	Credelio Plus^®^	135	5–100	11.8	–
	Afoxolaner + Milbemycin oxime	67	5–139	11.4	–
14 ± 2	Credelio Plus^®^	130	0–12	0.15	98.7
	Afoxolaner + Milbemycin oxime	66	0–6	0.17	98.5
28 ± 2	Credelio Plus^®^	132	0–3	0.05	99.6
	Afoxolaner + Milbemycin oxime	66	0–20	0.32	97.2
56 ± 2	Credelio Plus^®^	131	0–0	0	100
	Afoxolaner + Milbemycin oxime	65	0–3	0.05	99.6
84 ± 2	Credelio Plus^®^	126	0–0	0	100
	Afoxolaner + Milbemycin oxime	65	0–0	0	100

The majority of fleas found on study dogs were identified by the diagnostic laboratory as cat fleas (*Ctenocephalides felis*) followed by dog fleas (*C. canis*), human fleas (*Pulex irritans*), hedgehog fleas (*Archaeopsylla erinacei*) and hen fleas (*Ceratophyllus gallinae*). For cat fleas, dog fleas, and human fleas there were a sufficient number of cases (at least 5 cases with ≥ 5 live fleas per species on Day 0) to conduct separate analyses for these individual species (Table 3). By Day 28 after a single treatment Credelio Plus^®^ had reduced live flea counts for these three species by 99.6 to 100% and by Day 56 effectiveness was 100%. By Day 28 afoxolaner + milbemycin oxime had reduced live flea counts for these three species by 94.0% to 100% and by Day 56 effectiveness was 99 to 100% (Table 3).

**Table 3:** Effectiveness of three consecutive monthly doses of Credelio Plus^®^ and afoxolaner + milbemycin oxime based on laboratory counts and speciation of natural flea infestations for dog, cat and human flea species on client owned dogs in European field study

Study day	Treatment group	*C. felis*		*C. canis*		*P. irritans*	
		AM flea count	% Effectiveness	AM flea count	% Effectiveness	AM flea count	% Effectiveness
0	Credelio Plus®	18.5	–	11.4	–	6.6	–
	Afoxolaner + Milbemycin oxime	10.4	–	20.7	–	10.5	–
14 ± 2	Credelio Plus®	0.25	98.6	0	100	0	100
	Afoxolaner + Milbemycin oxime	0.09	99.1	0.09	99.6	0.06	99.4
28 ± 2	Credelio Plus®	0.07	99.6	0	100	0	100
	Afoxolaner + Milbemycin oxime	0.63	94.0	0	100	0	100
56 ± 2	Credelio Plus®	0	100	0	100	0	100
	Afoxolaner + Milbemycin oxime	0.10	99.0	0	100	0	100
84 ± 2	Credelio Plus®	0	100	0	100	0–0	100
	Afoxolaner + Milbemycin oxime	0	100	0	100	0–0	100

Before treatment administration on Day 0, the number of dogs in the Credelio Plus^®^ and afoxolaner + milbemycin oxime groups infested with each species was 58 and 32 for *C. felis*, 29 and 11 for *C. canis* and 18 and 10 for *P. irritans*, respectively

### FAD

On Day 0 the arithmetic mean (AM) total FAD scores (defined as the sum of the component scores) were 4.65 and 4.07 in the IP and CP groups, respectively. From Day 14 onwards the AM total FAD scores were < 1 in both treatment groups. On Day 84 the AM total FAD scores were similar, 0.15 in the IP group and 0.17 in the CP group. See Table 4 for summary statistics for the total FAD score and for each individual clinical sign component. At all assessed time points and based on statistical analyses of the total FAD scores, non-inferiority of Credelio Plus^®^ compared to Nexgard Spectra^®^ was demonstrated.

**Table 4:** Effectiveness of three consecutive monthly doses of Credelio Plus^®^ and afoxolaner + milbemycin oxime and impact on AM total and individual FAD clinical sign scores on dogs with natural flea infestations in client owned dogs in European field study

Study day	Treatment group	*n*	Total clinical signs score	Pruritus	Erythema	Scaling	Papules	Alopecia	Dermatitis/Pyodermatitis
0	Credelio Plus^®^	110	4.65	1.79	0.95	0.79	0.37	0.41	0.39
	Afoxolaner + Milbemycin oxime	55	4.07	1.64	0.80	0.75	0.38	0.24	0.27
14 ± 2	Credelio Plus^®^	110	0.89	0.28	0.09	0.26	0.02	0.18	0.06
	Afoxolaner + Milbemycin oxime	55	0.64	0.31	0.02	0.15	0.0	0.11	0.06
28 ± 2	Credelio Plus^®^	110	0.53	0.20	0.03	0.16	0.0	0.09	0.05
	Afoxolaner + Milbemycin oxime	55	0.49	0.16	0.05	0.13	0.0	0.04	0.11
56 ± 2	Credelio Plus^®^	110	0.25	0.08	0.03	0.08	0.02	0.02	0.02
	Afoxolaner + Milbemycin oxime	55	0.19	0.08	0.02	0.04	0.0	0.04	0.02
84 ± 2	Credelio Plus^®^	110	0.15	0.06	0.01	0.02	0.01	0.02	0.03
	Afoxolaner + Milbemycin oxime	55	0.17	0.06	0.02	0.08	0.0	0.0	0.02

#### Tick cohort

Arithmetic mean (AM) total live tick counts and percent effectiveness are summarised in Table 5. In the IP group the total AM live tick count was 4.6 on Day 0. At post-treatment time points AM tick counts were ≤ 0.08, indicating that effectiveness in the IP group exceeded 98% throughout the study. At Day 28 after the first IP treatment tick effectiveness was 99.3%. Since effectiveness was ≥ 90% for both the IP and CP, the IP was considered non-inferior in relation to CP at all time points. All post-treatment tick counts were significantly lower than baseline in both treatment groups (*p* < 0.001). The difference in success rates was also evaluated and for each dog success was defined as a percentage reduction from baseline of 90%. At least 97% of dogs were successfully treated at all time points in both IP and CP groups.

**Table 5:** Effectiveness of three consecutive monthly doses of Credelio Plus^®^ and afoxolaner + milbemycin oxime based on investigator counts of natural live tick infestations for all tick species on client owned dogs in European field study

Study day	Treatment group	*n*	Live tick counts		% Effectiveness
			Range	Arithmetic mean	
0	Credelio Plus^®^	147	3–14	4.6	–
	Afoxolaner + Milbemycin oxime	74	3–18	4.2	–
7 ± 2	Credelio Plus^®^	145	0–5	0.08	98.2
	Afoxolaner + Milbemycin oxime	71	0–1	0.03	99.3
14 ± 2	Credelio Plus^®^	147	0–1	0.01	99.8
	Afoxolaner + Milbemycin oxime	74	0–1	0.01	99.8
21 ± 2	Credelio Plus^®^	144	0–1	0.01	99.8
	Afoxolaner + Milbemycin oxime	73	0–1	0.01	99.8
28 ± 2	Credelio Plus^®^	147	0–2	0.03	99.3
	Afoxolaner + Milbemycin oxime	73	0–1	0.01	99.8
42 ± 2	Credelio Plus^®^	145	0–0	0	100
	Afoxolaner + Milbemycin oxime	72	0–0	0	100
56 ± 2	Credelio Plus^®^	143	0–0	0	100
	Afoxolaner + Milbemycin oxime	72	0–0	0	100
70 ± 2	Credelio Plus^®^	135	0–0	0	100
	Afoxolaner + Milbemycin oxime	72	0–0	0	100
84 ± 2	Credelio Plus^®^	127	0–0	0	100
	Afoxolaner + Milbemycin oxime	65	0–4	0.06	98.6

The majority of ticks found on study dogs were identified as castor bean ticks (*Ixodes ricinus*) followed by brown dog ticks (*R. sanguineus s. l.*). Other less common tick species identified in both the IP and CP groups at the Day 0 baseline tick counts included *Dermacentor reticulatus, Haemaphysalis concinna, Hyalomma marginatum, Ixodes canisuga* and *I. hexagonus*. Since a sufficient number of cases (at least 5 cases with ≥ 3 live attached ticks per species) were available for castor bean tick and brown dog tick, tick counts for these two species were analysed separately (Table 6). In the tick effectiveness population, 94 dogs in the IP group and 48 dogs in the CP group had castor bean ticks identified on Day 0. Live tick counts from Day 7 (inclusive) onwards were significantly lower than baseline counts and percent reduction was  ≥93.8%, indicating both treatments were effective against *I. ricinus*. In the tick effectiveness population, 32 dogs in the IP group and 19 dogs in the CP group had brown dog ticks identified on Day 0, respectively. Live tick counts from Day 7 (inclusive) onwards were significantly lower than baseline counts and percent reduction was ≥94.2% indicating both treatments were effective against *R. sanguineus* (*s.l.*).

**Table 6:** Effectiveness of three consecutive monthly doses of Credelio Plus^®^ and afoxolaner + milbemycin oxime based on laboratory counts and speciation of natural *Ixodes ricinus* and *R. sanguineus* (*s.l.*) tick infestations on client owned dogs in European field study

Study day	Treatment group	*I. ricinus*		*R. sanguineus*	
		AM tick count	% Effectiveness	AM tick count	% Effectiveness
0	Credelio Plus^®^	4.6	–	5.9	–
	Afoxolaner + Milbemycin oxime	4.2	–	5.6	–
7 ± 2	Credelio Plus^®^	0.28	93.9	0.34	94.2
	Afoxolaner + Milbemycin oxime	0.26	93.8	0	100
14 ± 2	Credelio Plus®	0.04	99.1	0	100
	Afoxolaner + Milbemycin oxime	0.04	99.0	0.32	94.3
21 ± 2	Credelio Plus^®^	0.05	98.9	0.09	98.5
	Afoxolaner + Milbemycin oxime	0.06	98.6	0.16	97.1
28 ± 2	Credelio Plus^®^	0.06	98.7	0	100
	Afoxolaner + Milbemycin oxime	0.04	99.0	0	100
42 ± 2	Credelio Plus^®^	0.02	99.6	0	100
	Afoxolaner + Milbemycin oxime	0	100	0.16	97.1
56 ± 2	Credelio Plus^®^	0.07	98.5	0	100
	Afoxolaner + Milbemycin oxime	0	100	0	100
70 ± 2	Credelio Plus^®^	0	100	0	100
	Afoxolaner + Milbemycin oxime	0.02	99.5	0	100
84 ± 2	Credelio Plus^®^	0.03	99.3	0	100
	Afoxolaner + Milbemycin oxime	0.10	99.8	0	100

Before treatment administration on Day 0, the number of dogs in the Credelio Plus^®^ and afoxolaner + milbemycin oxime groups infested with each species was 94 and 48 for *I. ricinus* and 32 and 19 for *R. sanguineus*, respectively

## Discussion

To develop a new broad-spectrum endectocidal drug product for oral use in dogs that have concurrent nematode infections and also require treatment for an ectoparasite infestation(s), lotilaner was combined with milbemycin oxime to form Credelio Plus^®^. In the flea cohort of this European field study, effectiveness over the 84-day study for all flea species found on the IP treated dogs was 98.7% to 100%. The persistent or residual effectiveness demonstrated over the 84-day study phase from the orally administered Credelio Plus^®^ treatments provided excellent flea effectiveness. The high level of effectiveness contributed to reducing or eliminating existing infestations as well as re-infestations over time for any newly emerged fleas from the contaminated environment in each pet’s household. This excellent flea effectiveness is similar to what has been reported for the lotilaner mono product (Credelio™) in laboratory studies with induced infestations as well as in clinical field studies with natural flea burdens [14, 18, 19]. Flea effectiveness results from this field study are similar to other studies that assessed an oral systemic isoxazoline or spinosyn containing mono- or combination drug products [18, 29–32]. Onset of action as well as speed of kill for adult fleas of the mono product of lotilaner (Credelio™) has been previously reported [11, 14]. Lotilaner, as the ectoparasiticide component of Credelio Plus^®^, previously demonstrated rapid onset of action against existing fleas infestations on dogs with demonstrated effectiveness at 8 h of 99.6% [11]. Also, rapid speed of kill of lotilaner was demonstrated at 8 h and 12 h post-infestation, with effectiveness > 99% and 100%, respectively, through Day 35 [14]. The rapid onset of flea adulticidal effectiveness of lotilaner contributes to breaking the flea lifecycle by killing newly acquired flea infestations before the female flea can start production of eggs that further contaminate the pets’ environment [15].

The high level of effectiveness and persistent residual flea effectiveness after each monthly IP treatment significantly reduced the number of adult fleas in this field study and as a direct result made a major contribution to the management of FAD [3]. FAD is a common dermatological condition seen globally in dogs presented to veterinary practices [33–35]. On Day 0 the AM total FAD scores were 4.65 and 4.07 in the IP and CP groups, respectively. From Day 14 onwards the AM total FAD scores were < 1 in both treatment groups. On Day 84 at the end of the study, the AM total FAD scores were similar, 0.15 in the IP group and 0.17 in the CP group. The reductions in the assessed clinical signs of FAD in dogs over the 84-day study were a direct effect associated with the adulticidal flea effectiveness that occurred following the monthly treatments with Credelio Plus^®^. Pruritus is a common clinical sign observed by dog owners in flea-allergic dogs [33–36]. In the present study, pruritus was the most common assessed baseline clinical sign of FAD and had the highest baseline clinical score of 1.79 in the IP group. By Day 84, the pruritus clinical sign score in the IP group had declined to 0.06. These improvements in the clinical signs of FAD as observed in dogs in this European field study using Credelio Plus^®^ are similar to previous field studies in dogs treated with Credelio™ conducted both in Europe and the USA [18, 19, 39].

In the tick cohort of this European field study, effectiveness for all tick species found on the IP treated dogs was 98.2 to 100%. This high and sustained tick effectiveness demonstrated over the 84-day study phase from monthly oral Credelio Plus^®^ treatments was similar regardless of the country, region within countries, the different tick species infesting the enrolled dogs and the size of the tick burdens. The excellent tick effectiveness is similar to the lotilaner mono product (Credelio™) that was assessed using laboratory-induced infestations using both European and North American tick species and also in European clinical field studies for dogs with natural tick burdens [13, 17, 20–23, 29, 30, 32]. Lotilaner was previously shown to have a rapid onset of activity in dogs after treatment against *I. ricinus* and began to kill these ticks on dogs within 4 h of treatment with 100% effectiveness within 8 h [17]. Lotilaner as the mono product (Credelio™) sustained a rapid kill of newly acquired infestations of *I. ricinus* through 35 days [17]. As this study noted, by quickly killing ticks that infest dogs, lotilaner has the potential to stop or reduce the transmission of tick-borne disease agents. Lotilaner was subsequently shown to prevent *Dermacentor reticulatus* transmission of *Babesia canis* to dogs [37].

Safety was also assessed in this study where 1264 doses of the IP combination product were administered to 427 dogs. Adverse events documented during the study were consistent with abnormal observations seen globally by pet owners and routinely seen in any general dog population and were not considered to be associated with the administration of the IP. The AE profile of the Credelio Plus^®^ was similar to what was seen with the CP (Nexgard Spectra^®^). Abnormal health observations documented in this field study were not unexpected as the single components of the combination product have been commonly used and/or are well characterised in dogs. MO used alone or in combination with other oral parasiticides has been used safely for over 20 years for intestinal nematode control and heartworm prevention in dogs [38]. Lotilaner (Credelio™) as a standalone oral ectoparasiticide has demonstrated safety for dogs under both field use and laboratory conditions [11–23, 39].

## Conclusions

This pivotal randomised, blinded, positive-controlled, multicentre, GCP-compliant, European field study demonstrated the effectiveness and safety of Credelio Plus^®^, a combination of lotilaner at a dose rate of 20.0–41.5 mg/kg bodyweight and milbemycin oxime at a dose rate of 0.75–1.53 mg/kg bodyweight administered orally to dogs naturally infested with fleas and/or ticks. Three consecutive monthly Credelio Plus^®^ treatments resulted in a 100% reduction of flea from Days 56 to 82 and for tick infestations from Day 42 to the end of the study on Day 84 and a substantial reduction in, or elimination of signs of FAD. This new authorised combination treatment option of lotilaner + MO (Credelio Plus^®^) offers immediate and persistent flea and tick treatment, treatment of larval and adult intestinal nematodes and heartworm and lungworm prevention and can be used to provide broad-spectrum parasite control for dogs (EMA/CVMP/65055/2021; ATCvet code: QP54AB51) [40]. This will contribute to owner compliance and the adherence to treatment recommendations from global scientific groups to prevent or treat these parasites as well as decreasing the transmission of important zoonotic parasites and tick- and flea-transmitted disease agents.

## Data Availability

The dataset summarizing and supporting the conclusions of this article are included within the article. Due to commercial confidentiality of the research, data not included in the manuscript can only be made available to bona fide researchers subject to a non-disclosure agreement.

## Abbreviations

AE: Adverse event
AM: Arithmetic mean
CP: Control veterinary product
ESCCAP: European Scientific Counsel Companion Animal Parasites
FAD: Flea allergy dermatitis
GCP: Good clinical practice
IP: Investigational veterinary product
MO: Milbemycin oxime
VICH: Veterinary International Conference on Harmonization
